# Supplementary material for: ABCG2 Gene Expression in Non-Small Cell Lung Cancer
Source: Biomedicines. 2024 Oct 19;12(10):2394. doi: 10.3390/biomedicines12102394 (PMC11504646; doi:10.3390/biomedicines12102394)

Figure S1. Association between *ABCG2* expression and clinicopathological characteristics, including **(A)** cancer stage, **(B)** nodal metastasis status, **(C)** smoking status and **(D)** *TP53* mutation status in LUAD and LUSC (* p < 0.05; UALCAN).

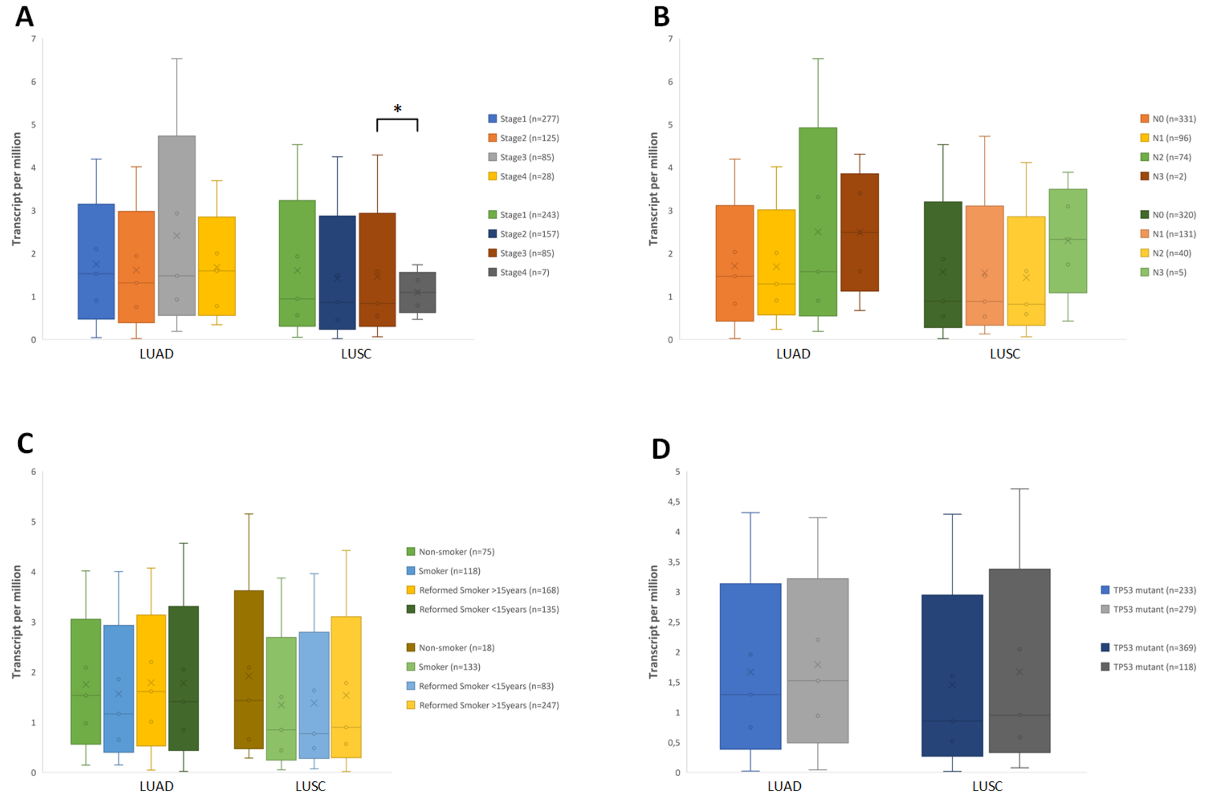

Figure S2. Association of *ABCG2* expression **(A)** with clinicopathological and demographic features, **(B)** and analysis of promoter region methylation in LUAD from TCGA datasets (n=861) (*** p < 0.001; MEXPRESS).

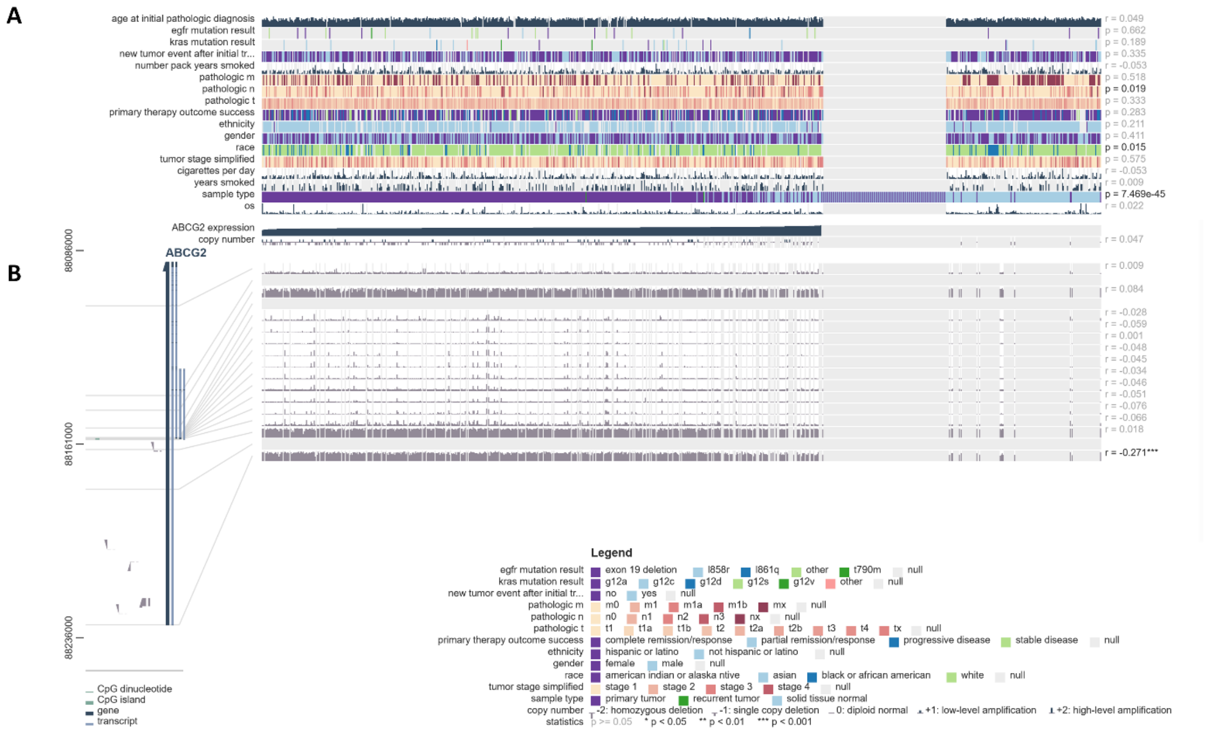

Figure S3. Association of *ABCG2* expression **(A)** with clinicopathological and demographic features, **(B)** and analysis of promoter region methylation in LUSC from TCGA datasets (n=758) (* p < 0.05; ** p < 0.01; *** p < 0.001; MEXPRESS).

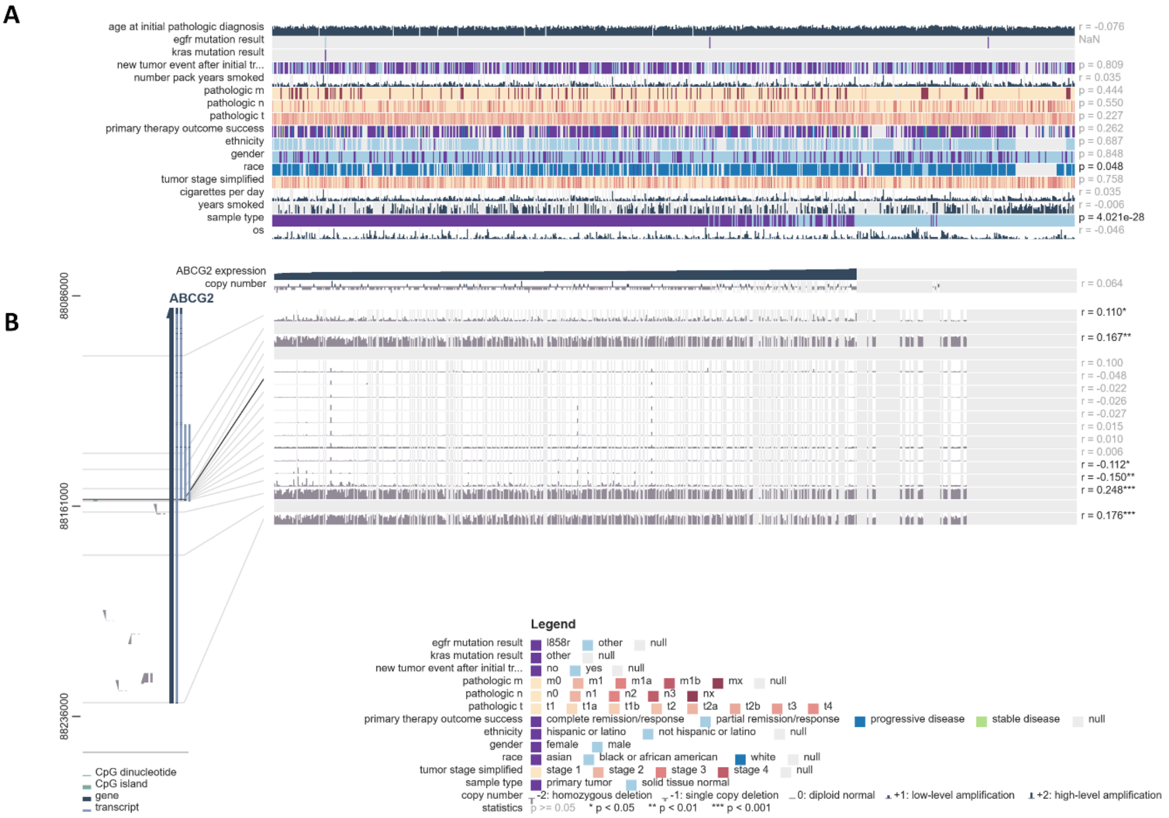

Figure S4. The correlation of *ABCG2* gene expression (low vs. high expression level) with overall survival (OS) in subgroups of patients with LUAD. The analysis included subgroups formed according to: cancer stage (**(A)** stage 1; **(B)** stage 2; **(C)** stage 3), tumor's grade (**(D)** G1; **(E)** G2; **(F)** G3), getting chemotherapy (**(G)** yes; **(H)** no), gender (**(I)** male; **(J)** female), tobacco smoking (**(K)** those who never smoked; **(L)** those who smoke or have smoked in the past). HR, hazard ratio; red: high expression; black: low expression ($p < 0.05$; Kaplan–Meier plotter)

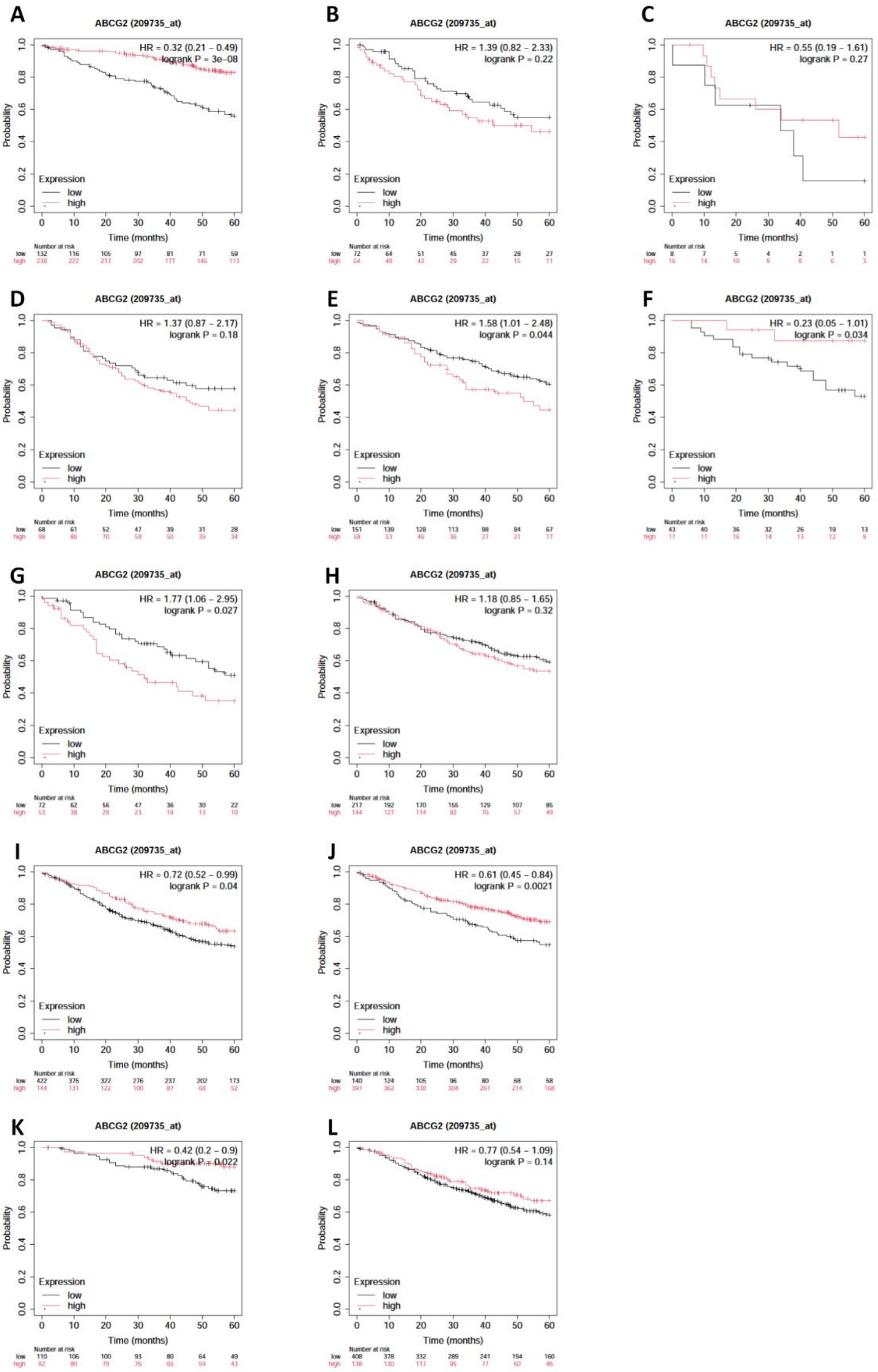

Figure S5. *ABCG2* gene expression levels in the blood of lung cancer patients depending on **(A)** age at diagnosis (p=0.1995), **(B)** histological type of the cancer (p=0.2638), **(C)** grade of histological malignancy (p=0.6334), **(D)** cancer stage (p=0.8486), **(E)** gender (p=0.8433) **(F)** and cigarette smoking (p=0.3604). **(G)** Comparison of relative *ABCG2* expression in blood taken from patients at three points during the diagnostic-therapeutic procedurę (p=0.8187).

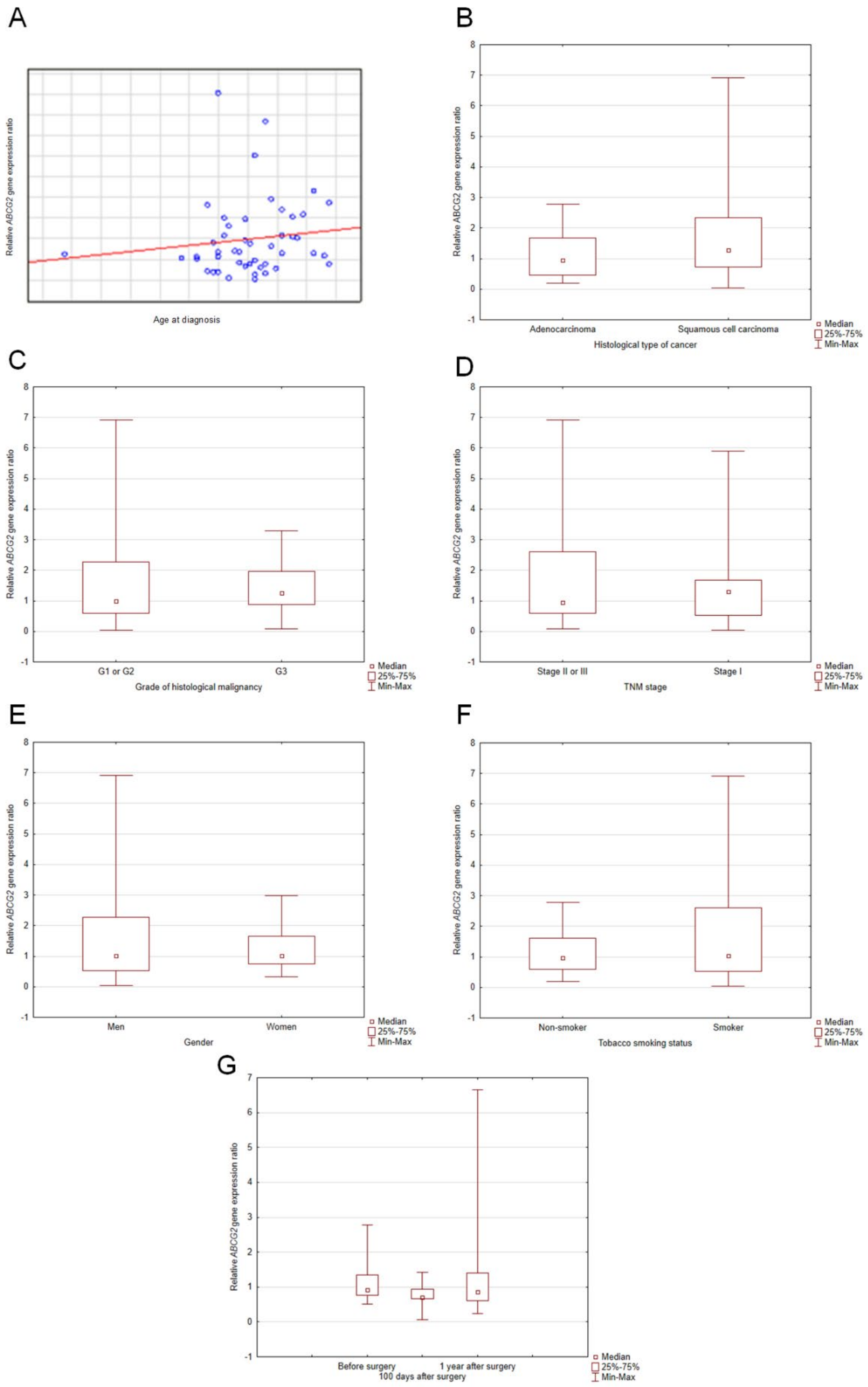

Supplement: Supplementary file 1 [file biomedicines-12-02394-s001.zip › biomedicines-3236257-supplementary.pdf]
